# Day length may make geographical difference in body size and proportions: An ecological analysis of Japanese children and adolescents

**DOI:** 10.1371/journal.pone.0210265

**Published:** 2019-01-22

**Authors:** Masana Yokoya, Yukito Higuchi

**Affiliations:** 1 Shimonoseki Junior College, Shimonoseki, Yamaguchi, Japan; 2 Kyushu Kyoritsu University, Yahatanishi-ku, Kitakyushu, Japan; TNO, NETHERLANDS

## Abstract

There is a north-south gradient in the body heights of Japanese children. A hypothesis had previously been proposed that differences in thyroid hormone activity induced by geographical differences in effective day length (duration of photoperiod exceeding a predetermined light intensity) might cause the differences in height. If thyroid hormone is involved, the effect should extend to body weight. This study examined whether geographical differences in body height and weight can be explained in terms of thyroid hormone activity induced by geographical differences in the photoperiodic environment using prefecture-level anatomical data and Japanese Mesh Climatic Data. Multiple regression analysis demonstrated that the combination of effective day length and weight was statistically significant as a predictor of height. Controlling for body weight revealed that effective day length was inversely correlated with height. Multiple regression analysis revealed that a combination of effective day length and height was statistically significant as a predictor of weight. Controlling for height demonstrated that effective day length was positively correlated with weight. Assuming an inverse correlation between effective day length and thyroid hormone activity, these results appear to show that short day-length will increase the activity of thyroid hormone and contribute to increasing height, but will inhibit weight gain; in contrast, long day-length will decrease the activity of thyroid hormone and contribute to increasing weight but will inhibit height gain. Geographical differences in height, and weight, and part of the prevalence of obesity in Japanese children and early adolescents may be explained by geographical differences in effective day length.

## Introduction

The height of Japanese children raised in the northern region is greater than that of children raised in southern regions, creating a north-south gradient in physique [1–4]. This tendency can be clearly seen at least for the last 50 years of school health statistics of Japan [5–6]. In the last 50 years, the physique of children throughout Japan has improved. Nevertheless, the north-south gradient in physique still remains. After consideration of the nutritional improvements, economic growth, and intense migration that occurred during the past 50 years throughout Japan, the north-south gradient in physique is probably the result of environmental rather than nutritional or genetic factors [1].

Previous studies have shown that geographical differences in the height of Japanese children correspond well with the distribution of effective day length [1]. Effective day length takes into account the intensity of illuminance. Although the annual possible sunshine duration is the same at all points of the globe, the distribution of effective day length becomes shorter with decreases in solar radiation [1,7]. The height of Japanese children tends to be higher in areas where the effective day length is short [1].

In connection with this, there are studies reporting that height growth is accelerated in the summer season when the day length is greater. In Japan, a big-data survey using a smart phone application indicated that the heights of children below the age of 1 are likely to increase in the summer [8]. Moreover, several studies on children with growth hormone deficiency receiving continuous exogenous recombinant human growth hormone (r-hGH) therapy have shown that the results of growth hormone treatment display seasonality and latitudinal differences [9–11]. As the consequences of r-hGH treatment become prominent in the summer season in higher latitudes, there appears to be a close relationship between height growth and day length [9].

However, there are inconsistencies in the relationship between the seasonality of height growth acceleration and the geographical distribution of height. Since height growth is accelerated during the summer, the distribution of heights is expected to mirror the distribution of summer day lengths; however, the height distribution of Japanese children is in good agreement with the distribution of winter or the annual mean effective day lengths rather than that of summer [12]. In order to account for this inconsistency, summer height velocity should be greater according to the shorter winter day length [12].

One of the metabolic pathways that may account for these observations comprises epigenetic modifications by reversible DNA methylation and thyroid hormone activity, which was examined in the reproductive system of seasonal breeding vertebrates [12–13]. For example, Siberian hamsters can adjust their reproductive timing in the spring according to the winter day length [13]. If this mechanism is applicable to humans, height growth acceleration in the summer will occur in accordance with short winter day length, and the distribution of height will be greater in areas where the day length is shorter. Moreover, height growth acceleration in the summer in response to r-hGH therapy can be explained by the synergistic effect of thyroid hormone and growth hormone [12].

If the seasonality of height growth and geographical differences in height are due to thyroid hormone activity, it should also influence the seasonality of weight gain and geographical differences in body weight. Thyroid hormone activity is thought to influence the seasonal fluctuation of appetite and body weight of both of humans and animals. Seasonal fluctuations in body weight are inversely correlated with fluctuations in height, and there are reports that body weight tends to increase in winter [14–17]. Moreover, the effect of thyroid hormone activity on body weight contrasts with that on height: if thyroid hormone expression is highly activated, metabolism increases and body weight decreases; if thyroid hormone is highly suppressed, metabolism decreases and body weight increases. Given that thyroid hormone activity affects geographical differences in height, geographical differences in body weight should also be thought of as being related to height.

The aim of this study was to elucidate the influence of the photoperiodic environment on geographical differences in height and weight among Japanese children and adolescents focusing on thyroid hormone activity. In this study, we investigated the geographical association between both body height and weight and effective day length in Japanese children and adolescents, using precise climatic data and the Geographic Information Science (GIS) technique. Moreover, we discussed whether geographical differences in body height and weight could be explained in terms of geographical differences in thyroid hormone activity induced by the photoperiodic environment.

## Materials and methods

### Study area

This ecological study was conducted using prefecture-level data from Japanese children and adolescents. S1 Fig shows a map of the 47 prefectures in Japan [1]. Each prefecture was assigned a number (S1–S4 Tables).

### Anatomical data

Prefecture-level anatomical data on Japanese children and adolescents were collected from School Health Examination Surveys conducted between 1989 and 2013 by the Ministry of Education, Culture, Sports, Science, and Technology [5,6]. These surveys cover each of the 47 prefectures in Japan and contain data on mean height, weight, and other physical conditions, categorized according to sex and age (covering children between the ages of 5 and 17). Sample size and original profiles are included in this database [5,6].

In order to reduce accidental error and consider geographical differences, 25-year (1989–2013) means of standardized height and weight were calculated for each sex and age category using the following formula:

$$Y_{ij}=\frac{1}{25}\sum_{k=1}^{25}\frac{Z_{ijk}-u_{jk}}{\sigma_{jk}}$$ (1)

Where *i* is the prefecture, *j* is the group (defined by age and sex), *k* is the year (1989–2013), *Yij* are the standardized data over the 25-year period for each prefecture and sex standardized by mean, *Zij* represents the prefecture data, *μjk* is the national mean, and *σjk* is the national-level standard deviation based on the entire set of measurements obtained from the survey reports [5].

The standardized heights and weights averaged for the 25-year period for each prefecture are listed in S1 and S2 Tables. The associations between standardized height and weight according to age are shown in S3 Table.

### Climatic data

Since humans spend the majority of their time indoors, the dark period is prolonged more than in the outdoor natural environment [18–20]. This effect becomes more pronounced in high latitude regions experiencing a relatively weak intensity of daylight.

In a previous study, the concept of effective day length was proposed as a climatic element quantifying ambient daylight intensity and length [7]. Effective day length is the photoperiod that takes into account the intensity of light; it becomes shorter with decreases in solar radiation. The effective day length at 0 lux light intensity is equivalent to the possible sunshine duration. The effective day length at more than 1000 lux increases in almost direct proportion to the intensity of solar radiation in the data range observed in the area of Japan, and any effective day length is almost directly proportional to another at its intensity exceeding 1000 lux. Monthly means of effective day length at any light intensity can be calculated by the empirical model using monthly mean solar radiation data [7].

Mesh Climatic Data 2000 [21] were used to compare geographical differences in height and weight. Mesh Climatic Data 2000 is a climatological standard which represents the average climate over 30 years from 1971 to 2000, and has been developed by aggregating the observed data of each Japanese meteorological station taking into consideration topographical factors. Mesh Climatic Data 2000 includes data such as monthly mean solar radiation with 1-km^2^ grid resolution. The monthly mean solar radiation data were used as inputs to calculate the monthly value of the effective day length.

Since any effective day length is almost directly proportional to another at its intensity exceeding 1000 lux, 5000 lux was set as a convenient threshold for the effective day length in this analysis. Since the distribution of solar radiation and any threshold of effective day length are almost proportional to each other, the threshold of effective day length becomes longer in regions receiving greater solar radiation in terms of probability. Therefore, the individual level differences are offset in a population-based study.

First, monthly means of effective day length at 5000 lux were calculated. Second, the annual mean of the effective day length (*Yavg*) was calculated using the following formula:

$$Yavg=\frac{\sum \left(M\times d\right)}{\sum \left(d\right)}$$ (2)

Where *M* is the monthly average of the effective day length, and *d* is the number of days in the month.

Because average resident populations differ from mesh to mesh, it is undesirable to obtain mean prefecture effective day lengths based on a simple calculation of the mean mesh value. To account for spatial differences between populations, Mesh Population Data from 2005, which were compiled from the results of the 2005 Population Census [22] and produced under the same code and standards as the Mesh Climatic Data 2000, were used to calculate the population-weighted prefectural mean effective day length (*Eavg*) for each prefecture using the following formula:

$$Eavg=\frac{\sum_{n=1}^{m}\left(T\times P\right)}{\sum_{n=1}^{m}\left(P\right)}$$ (3)

Where *m* is the number of meshes in each prefecture, *T* is the effective day length in the mesh, and *P* is the population density of the mesh. The population-weighted mean effective day length at 5000 lux derived from the mesh data of each prefecture is listed in S4 Table.

### Data analysis

Correlation analysis was carried out using standardized height and weight data and population-weighted climatic value data (annual mean effective day length) for all 47 prefectures. The association between anatomical data and effective day length was further explored via multiple linear regression modeling.

In a previous study it was rekported that the following model became highly statistically significant in early adolescence [1]:

$$H_{i}=k_{1}W_{i}-k_{2}E_{i},$$ (4)

where *H* is the linear predictor of the mean height for each prefecture in area *i*, *W* is the mean weight for each prefecture in area *i*, *E* is the population-weighted effective day length in area *i*, and *k*_*1*_ and *k*_*2*_ are the standardized regression coefficients representing the effects of *W* and *E*, respectively. In this model, weight was included to remove any biases caused by geographical differences in nourishment, assuming that weight is an index of food intake [1]. In this model, effective day length was a negative predictor of height.

By modifying Eq (4) the following equation of body weight can be obtained:

$$W_{i}=k_{1}H_{i}+k_{2}E_{i},$$ (5)

In this model, the effective day length is a positive predictor of weight.

Both multiple regression models (4) and (5) were applied to men and women between 5 and 17 years of age.

All statistical analyses were carried out using R version 3.0.2 [23].

## Results

**Fig 1:**
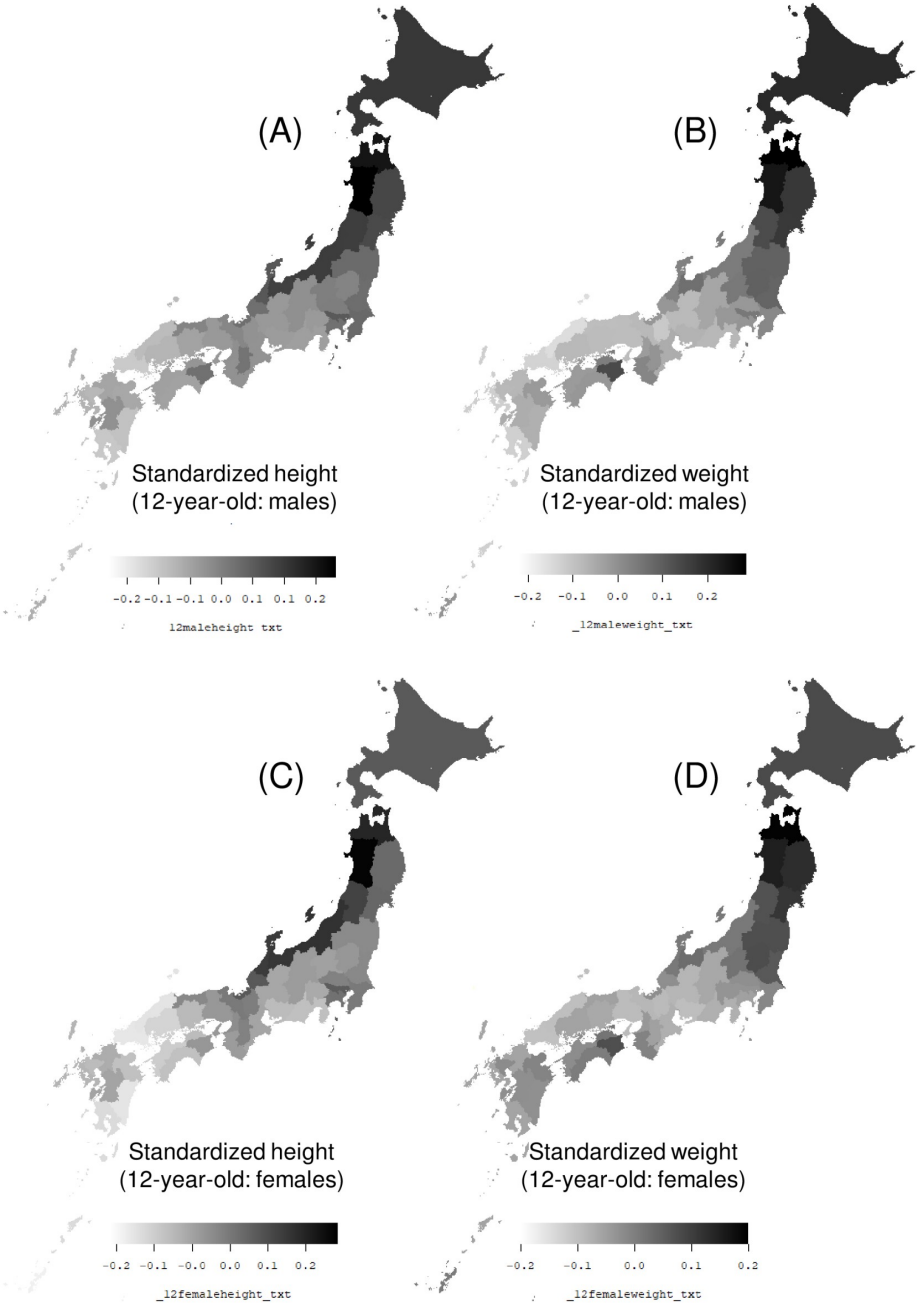
Map of the distribution of standardized heights and weights of Japanese youth. Map of the distribution of the 25-year (1989 to 2013) average of standardized heights and weights of the following subject groups, in each prefecture: (A) Heights of 12-year-old boys, (B) weights of 12-year-old boys, (C) heights of 12-year-old girls, and (D) weights of 12-year-old girls.

Fig 1 presents a map of the distribution of mean standardized heights and weights of 12-year-old Japanese boys and girls over the 25-year study period. Heights tend to be greater in northern Japan and northern areas along the coast of the Sea of Japan. Weights also tend to be greater in Northern Japan; however, the weight distribution displays a zonal pattern (parallel to latitude).

Table 1 presents basic statistics regarding the heights and weights of Japanese youth standardized over a 25-year period from 1989 to 2013. The maximum heights were observed in north Japan (Akita, Aomori), and the minimum heights were observed in south Japan (Okinawa, Miyazaki, and Yamaguchi). Similarly, the maximum weights were observed in north Japan (Aomori and Akita), and the minimum weights were observed in south Japan (Okinawa, Yamaguchi, and Shimane).

**Table 1:** Basic statistics for standardized heights and weights.

Standardized Height										
	Boys					Girls				
Age	5	8	11	14	17	5	8	11	14	17
Maximum	0.214	0.218	0.237	0.219	0.166	0.221	0.223	0.226	0.158	0.121
	Akita	Akita	Akita	Akita	Akita	Akita	Aomori	Aomori	Akita	Akita
Minimum	-0.261	-0.246	-0.135	-0.171	-0.305	-0.202	-0.133	-0.100	-0.306	-0.323
	Okinawa	Okinawa	Yamaguchi	Miyazaki	Okinawa	Okinawa	Okinawa	Yamaguchi	Okinawa	Okinawa
Mean	-0.009	-0.006	-0.001	-0.008	-0.011	-0.003	0.001	0.004	-0.012	-0.013
Median	-0.014	0.000	-0.028	-0.018	-0.008	-0.010	-0.007	-0.006	-0.020	-0.007
Standardized Weight										
	Boys					Girls				
Age	5	8	11	14	17	5	8	11	14	17
Maximum	0.234	0.295	0.289	0.262	0.220	0.242	0.293	0.255	0.229	0.195
	Aomori	Aomori	Aomori	Aomori	Akita	Aomori	Aomori	Aomori	Aomori	Akita
Minimum	-0.133	-0.106	-0.157	-0.158	-0.139	-0.135	-0.099	-0.099	-0.107	-0.254
	Okinawa	Yamaguchi	Shimane	Yamaguchi	Yamaguchi	Okinawa	Yamaguchi	Yamaguchi	Okinawa	Okinawa
Mean	-0.001	0.008	0.003	-0.002	0.002	0.003	0.013	0.011	0.011	0.014
Median	-0.031	-0.026	-0.030	-0.025	-0.016	-0.031	-0.014	-0.008	-0.015	0.002

**Fig 2:**
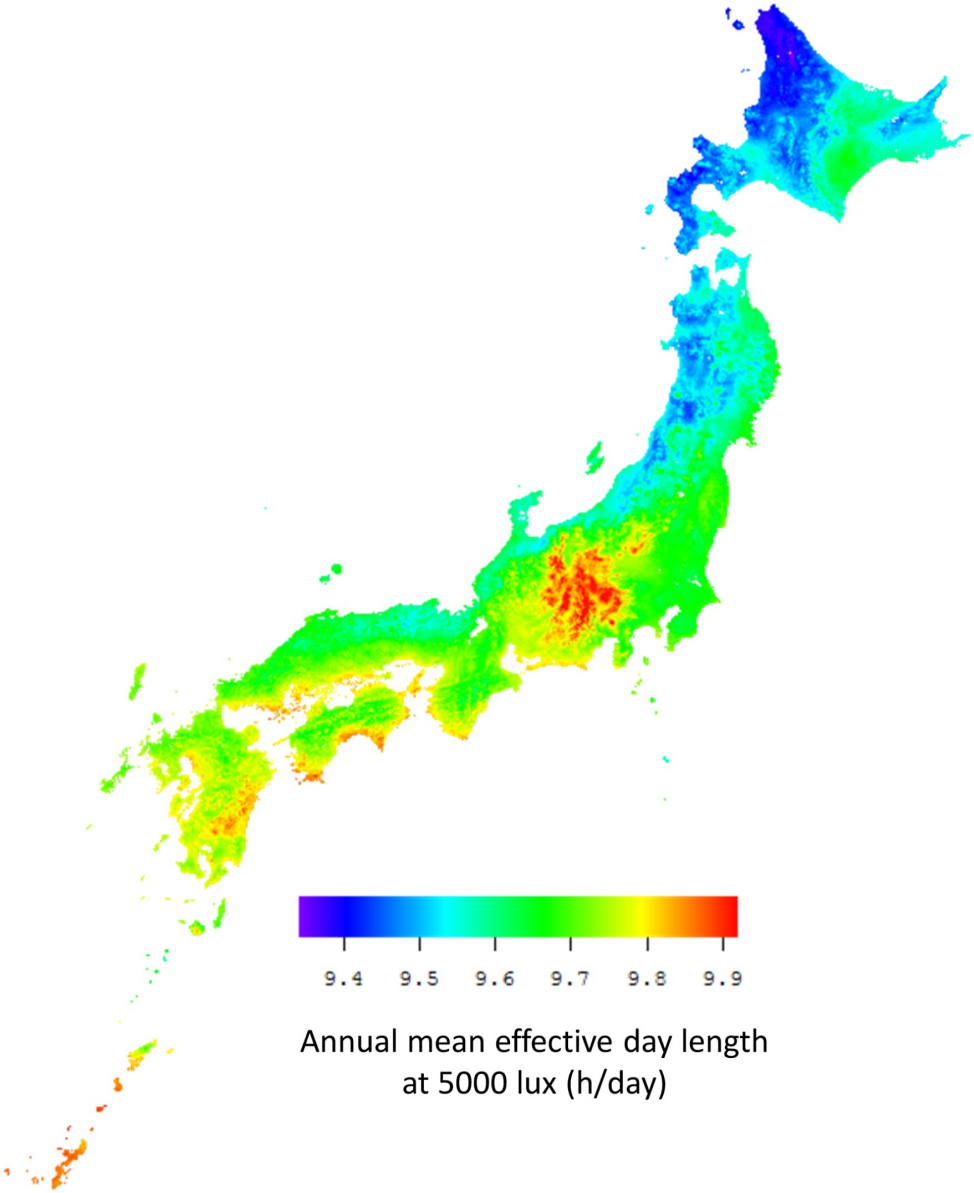
Map of the distribution map of effective day length in Japan. Mesh Climatic Data 2000 for 380,000 1-km^2^ mesh areas are shown. The fill color in the mesh areas indicates the annual mean effective day length at 5000 lux (h/day). The latter tend to be relatively low in northern areas along the Sea of Japan.

Fig 2 presents the distribution of the annual mean effective day length at 5000 lux (h/day) in Japan, which was extracted from Japanese Mesh Climatic Data 2000 for 380,000 1-km^2^ mesh areas. The annual mean effective day length at 5000 lux tended to be relatively low in the northern areas along the Sea of Japan.

Table 2 presents the statistics for the 30-year average population-weighted effective day length at 5000 lux. The maximum annual mean effective day length at 5000 lux was observed in Koch. The minimum annual mean effective day length at 5000 lux was observed in Akita.

**Table 2:** Basic statistics for climatic variables.

	Annual mean effective day length at 5000 lux (h/day)
Maximum	10.50 (Kochi)
Minimum	10.03 (Akita)
Mean	10.28
Median	10.30

Table 3 presents the Pearson’s correlation coefficients for the relationship between the heights and weights of 5- to 17-year-olds and the annual mean effective day length at 5000 lux. Height was significantly negatively correlated with effective day length at 5000 lux for all ages. The correlation between height and effective day length tended to be stronger in early adolescence for both boys and girls (14-year-old boys: r = -0.89, 12-year-old girls: r = -0.88).

**Table 3:** Pearson’s correlation coefficients for the relationship between height, weight, and effective day length.

	Height vs. effective day length	Weight vs. effective day length	Height vs. weight
Boys			
Age: 5	-0.81**	-0.54**	0.82**
6	-0.79**	-0.57**	0.82**
7	-0.82**	-0.61**	0.82**
8	-0.82**	-0.61**	0.81**
9	-0.84**	-0.61**	0.83**
10	-0.82**	-0.58**	0.86**
11	-0.80**	-0.54**	0.88**
12	-0.81**	-0.52*	0.87**
13	-0.86**	-0.55**	0.84**
14	-0.89**	-0.60**	0.78**
15	-0.87**	-0.61**	0.68**
16	-0.85**	-0.64**	0.66**
17	-0.83**	-0.66**	0.63**
Girls			
Age: 5	-0.80**	-0.53*	0.84**
6	-0.78**	-0.57**	0.84**
7	-0.83**	-0.61**	0.83**
8	-0.84**	-0.60**	0.85**
9	-0.83**	-0.57**	0.86**
10	-0.81**	-0.53*	0.86**
11	-0.83**	-0.48*	0.78**
12	-0.88**	-0.43*	0.58**
13	-0.82**	-0.50*	0.49*
14	-0.84**	-0.53*	0.48*
15	-0.79**	-0.55**	0.49*
16	-0.76**	-0.58**	0.53*
17	-0.79**	-0.58**	0.56**

**p < 0.0001.

*p < 0.005.

Weight and effective day length at 5000 lux were negatively correlated for all ages. However, the correlations were weaker than those between height and effective day length at 5000 lux. The correlations between weight and effective day length tended to weaken in early adolescence in both boys and girls (12-year-old boys: r = -0.52, 12-year-old girls: r = -0.43).

Height and weight were significantly correlated for all ages in both sexes. The correlation between height and weight was maximal in preteens (11-year-old boys: r = 0.88, 9–10-year-old girls: r = 0.86) and gradually weakened towards late adolescence.

Table 4 presents the results of multiple linear regression analyses performed to predict the height of 5- to 17-year-old Japanese boys. The results indicate that the combination of weight and annual mean effective day length at 5000 lux was a significant predictor of height in childhood to early adolescence. Annual mean effective day length was negatively correlated with height. This means that if weight is controlled for, body height increases with shortening effective day length. The accuracy of the multiple regression model reached the maximum in early adolescence. Predictive power decreased and weight was no longer a significant predictor in late adolescence.

**Table 4:** Regression coefficients (standard errors) of predictors of height (boys).

Boys	Predictors	Regression coefficient	Standard error	95% CI Lower	95% CI Upper	t	p	Adjusted R^2^
Age:5	Weight	0.53	0.07	0.40	0.66	8.03	<0.001	0.836
	Ann5000	-0.53	0.07	-0.66	-0.39	-7.94	<0.001	
7	Weight	0.51	0.08	0.35	0.66	6.48	<0.001	0.803
	Ann5000	-0.51	0.08	-0.67	-0.35	-6.50	<0.001	
9	Weight	0.51	0.07	0.38	0.65	7.48	<0.001	0.842
	Ann5000	-0.52	0.07	-0.66	-0.39	-7.62	<0.001	
11	Weight	0.63	0.05	0.53	0.73	12.68	<0.001	0.898
	Ann5000	-0.46	0.05	-0.56	-0.36	-9.38	<0.001	
13	Weight	0.53	0.05	0.43	0.62	11.07	<0.001	0.906
	Ann5000	-0.57	0.05	-0.66	-0.47	-11.96	<0.001	
15	Weight	0.24	0.08	0.07	0.41	2.87	0.006	0.769
	Ann5000	-0.72	0.08	-0.89	-0.55	-8.53	<0.001	
17	Weight	0.14	0.11	-0.08	0.36	1.31	0.198	0.675
	Ann5000	-0.74	0.11	-0.96	-0.52	-6.81	<0.001	

Ann5000: Annual mean effective day length at 5000 lux

Table 5 presents the results of multiple linear regression analyses performed to predict the height of 5- to 17-year-old Japanese girls. The results indicate that the combination of weight and annual mean effective day length at 5000 lux was a significant predictor of height in childhood to early adolescence. Annual mean effective day length at 5000 lux was negatively correlated with height as a predictor. This means that if weight is controlled for, body height increases with shortening effective day length. The accuracy of the multiple regression model reached a maximum for 9-year-olds, and this was earlier than for boys. Predictive power decreased after that and weight was no longer a significant predictor in adolescence.

**Table 5:** Regression coefficients (standard errors) of predictors of height (girls).

Girls	Predictors	Regression coefficient	Standard error	95% CI Lower	95% CI Upper	t	p	Adjusted R^2^
Age:5	Weight	0.59	0.06	0.46	0.71	9.67	<0.001	0.857
	Ann5000	-0.49	0.06	-0.61	-0.36	-8.02	<0.001	
7	Weight	0.51	0.07	0.37	0.66	7.17	<0.001	0.830
	Ann5000	-0.52	0.07	-0.66	-0.37	-7.19	<0.001	
9	Weight	0.57	0.06	0.46	0.68	10.37	<0.001	0.884
	Ann5000	-0.50	0.06	-0.61	-0.39	-9.08	<0.001	
11	Weight	0.49	0.06	0.38	0.61	8.52	<0.001	0.859
	Ann5000	-0.60	0.06	-0.72	-0.48	-10.39	<0.001	
13	Weight	0.11	0.10	-0.09	0.30	1.08	0.284	0.651
	Ann5000	-0.77	0.10	-0.96	-0.57	-7.88	<0.001	
15	Weight	0.08	0.11	-0.14	0.29	0.71	0.479	0.605
	Ann5000	-0.75	0.11	-0.97	-0.54	-7.01	<0.001	
17	Weight	0.16	0.11	-0.07	0.38	1.41	0.164	0.603
	Ann5000	-0.69	0.11	-0.92	-0.47	-6.27	<0.001	

Ann5000: Annual mean effective day length at 5000 lux

Table 6 presents the results of multiple linear regression analyses performed to predict the weight of 5- to 17-year-old Japanese boys. The results indicate that the combination of height and annual mean effective day length at 5000 lux was a significant predictor of weight in early adolescence. This means that if height is controlled for, body weight increases with greater effective day length. Effective day length at 5000 lux was positively correlated with weight as a predictor. The accuracy of the multiple regression model reached its maximum for 11-year-olds.

**Table 6:** Regression coefficients (standard errors) of predictors of weight (boys).

Boys	Predictors	Regression coefficient	Standard error	95% CI Lower	95% CI Upper	t	p	Adjusted R^2^
Age:5	Height	1.11	0.14	0.83	1.39	8.03	<0.001	0.682
	Ann5000	0.36	0.14	0.08	0.64	2.60	0.013	
7	Height	0.95	0.15	0.66	1.25	6.48	<0.001	0.648
	Ann5000	0.17	0.15	-0.13	0.46	1.14	0.262	
9	Height	1.08	0.14	0.79	1.37	7.48	<0.001	0.693
	Ann5000	0.29	0.14	0.00	0.58	2.02	0.049	
11	Height	1.25	0.10	1.05	1.44	12.68	<0.001	0.820
	Ann5000	0.46	0.10	0.26	0.66	4.68	<0.001	
13	Height	1.39	0.13	1.14	1.64	11.07	<0.001	0.787
	Ann5000	0.64	0.13	0.39	0.90	5.11	<0.001	
15	Height	0.64	0.22	0.19	1.08	2.87	0.006	0.433
	Ann5000	-0.05	0.22	-0.50	0.39	-0.24	0.813	
17	Height	0.26	0.20	-0.14	0.66	1.31	0.198	0.426
	Ann5000	-0.45	0.20	-0.85	-0.05	-2.27	0.028	

Ann5000: Annual mean effective day length at 5000 lux

Table 7 presents the results of multiple linear regression analyses performed to predict the weight of 5 to 17-year-old Japanese girls. The results indicate that the combination of height and annual mean effective day length at 5000 lux was a significant predictor of weight in early adolescence. This means that if height is controlled for, body weight increases with greater effective day length. Effective day length at 5000 lux was positively correlated with weight as a predictor. The accuracy of the multiple regression model reached a maximum with 9-year-olds and this was earlier than for boys.

**Table 7:** Regression coefficients (standard errors) of predictors of weight (girls).

Girls	Predictors	Regression coefficient	Standard error	95% CI Lower	95% CI Upper	t	p	Adjusted R^2^
Age:5	Height	1.15	0.12	0.91	1.39	9.67	<0.001	0.740
	Ann5000	0.39	0.12	0.15	0.63	3.24	0.002	
7	Height	1.04	0.14	0.75	1.33	7.17	<0.001	0.680
	Ann5000	0.25	0.14	-0.04	0.54	1.72	0.092	
9	Height	1.23	0.12	0.99	1.47	10.37	<0.001	0.775
	Ann5000	0.45	0.12	0.21	0.69	3.78	<0.001	
11	Height	1.25	0.15	0.96	1.55	8.52	<0.001	0.675
	Ann5000	0.57	0.15	0.27	0.87	3.88	<0.001	
13	Height	0.24	0.22	-0.21	0.69	1.08	0.284	0.229
	Ann5000	-0.30	0.22	-0.75	0.15	-1.35	0.184	
15	Height	0.15	0.20	-0.27	0.56	0.71	0.479	0.268
	Ann5000	-0.43	0.20	-0.84	-0.02	-2.10	0.041	
17	Height	0.27	0.19	-0.12	0.66	1.41	0.164	0.329
	Ann5000	-0.37	0.19	-0.75	0.02	-1.91	0.062	

Ann5000: Annual mean effective day length at 5000 lux

**Fig 3:**
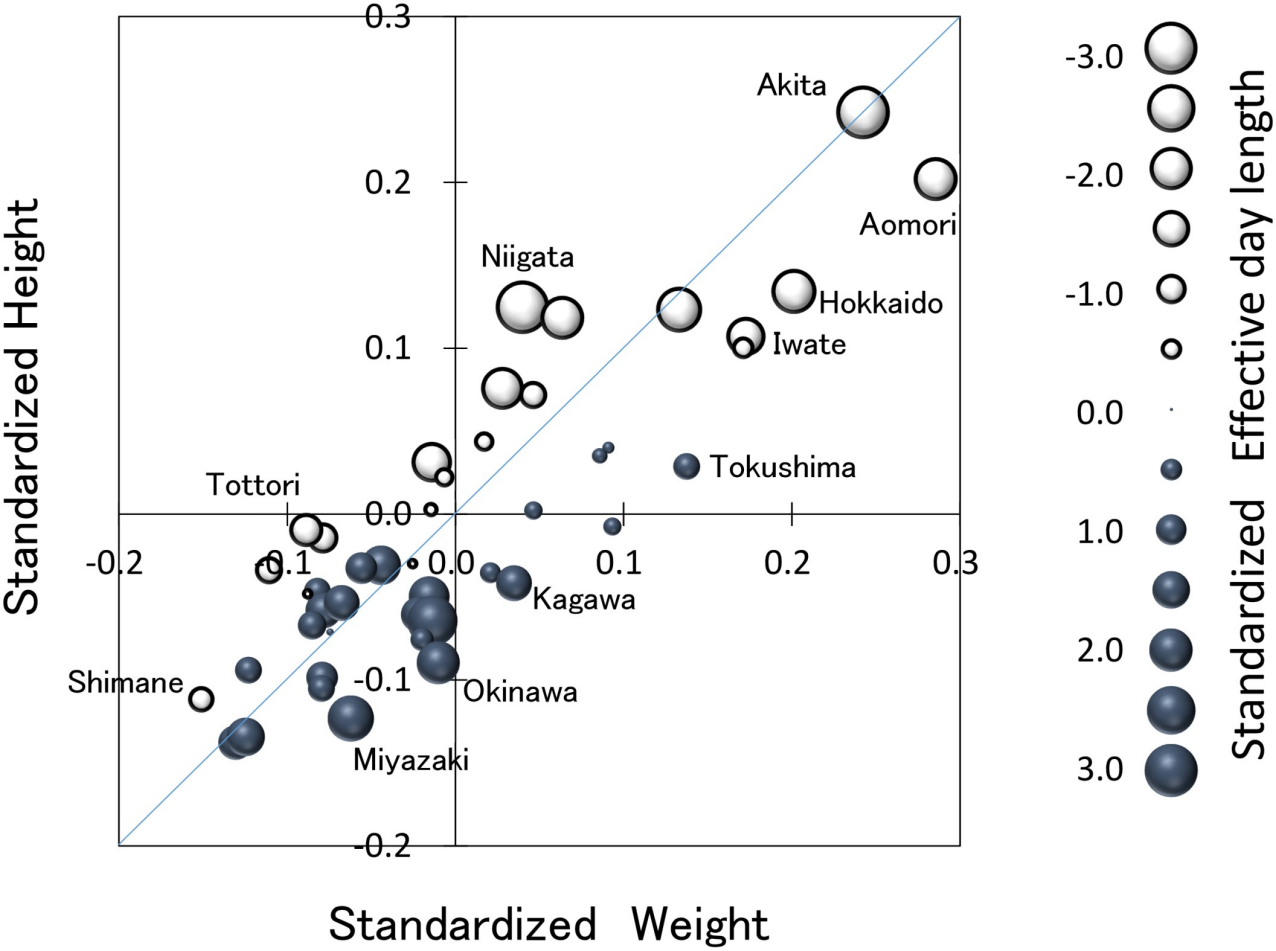
Relationship between standardized heights and weights. The relationship between standardized heights and weights in 12-year-old boys. The length of the standardized annual mean effective day length at 5000 lux is indicated by the size of the plot.

Fig 3 displays the relationship between standardized height and weight in 12-year-old boys over the 25-year study period. The standardized annual mean effective day length at 5000 lux is indicated by the size of the plot. Both height and weight increase when the effective day length is short. For the same weight, the shorter the day length, the higher the height becomes. For the same height, the longer the day length, the greater the weight becomes.

## Discussion

While many studies show that the height of individuals increases with increasing summer day length, the geographical distribution of the heights of Japanese children displays the opposite trend; the average height of Japanese children is greater in areas where the annual mean effective day length is shorter. In order to deal with this inconsistency, summer height velocity should be greater according to shorter winter day length [12]. One of the metabolic pathways that can be invoked to explain this phenomenon is involved in the epigenetic regulation of seasonal reproduction in mammals [12].

Recently, it was reported that seasonal reproductive processes in Siberian hamsters are controlled by reversible DNA methylation and thyroid hormone activity. In long-day breeding hamsters, exposure to short winter day lengths led to decreasing DNA methylation in the proximal promoter region of *Dio3* (Deiodinase Type III, which catabolizes thyroid hormone T4 to the inactive enantiomer rT3) and increased hypothalamic *Dio3* expression. De-methylation during the short winter day length was reversed, and methylation was promoted in anticipation of spring, as showed by re-methylation of the *Dio3* promoter and reduction in *Dio3* mRNA, resulting in spontaneous reproductive development. Short winter day length and winter like melatonin inhibited DNA methyltransferase expression and reduced *Dio3* promoter DNA methylation [13]. This means that the greater the de-methylation induced by short winter day length, the more pronounced the re-methylation and thyroid hormone activation in the spring. This mechanism lends support to the idea that quantitative information imparted by the short day length in winter is retained until summer [12].

If this mechanism is applicable to humans, height growth acceleration in the summer will occur in accordance with short winter day length, and the distribution of height will be greater in areas where day length is shorter. Moreover, height growth acceleration in the summer in response to r-hGH therapy can be explained by the synergistic effect of thyroid hormone and growth hormone [12].

In this study, the negative geographical correlation between effective day length and height was stronger towards adolescence; however, the correlation weakened towards late adolescence. This is probably because growth hormone and thyroid hormone play a significant role in growth before adolescence, whereas sex hormones become dominant after adolescence [24].

In correlation analyses, effective day length was independently negatively correlated with height and weight. In contrast, in multiple regression analyses for height estimation, the effective day length was a negative predictor in combination with weight; however, in multiple regression analyses for weight estimation, the effective day length was a positive predictor in combination with height. This indicates that the influence of the effective day length on weight is positive when considering the weight gain associated with height growth. Assuming that thyroid hormone activity is negatively linked to effective day length, these results can be explained by the assumption that thyroid hormone acts synergistically with growth hormone on height growth and thyroid hormone acts positively on weight gain when it is suppressed [14–17,24].

In multiple regression analyses, the relationship between height and weight becomes proportional, with the exception of obese or underweight individuals, and effective day length should not be chosen as a significant predictor. Formula (4) and (5) will not produce significant results unless height and weight, which are predictors other than with effective day length, are also influenced by the effective day length.

In other words, geographical differences in Japanese physique and deviation in the height-weight relationship seem to be caused by the influence of effective day length on height and weight. Effective day length negatively affects height (when being underweight is controlled for), and positively affects body weight (when being under-height is controlled for), resulting in deviations in the proportional relationship between height and weight. Therefore, not only height but also weight is affected by the effective day length.

In a previous study, geographical differences in climatic variables, such as temperature, solar radiation, and effective day length, were analyzed, and effective day length was selected as the primary negative predictor of the geographical gradient in body height when being underweight is controlled for [1]. In addition, it was shown by this study that effective day length is also a significant predictor of body weight when being under-height is controlled for. As predictors that are simultaneously significant for height and weight with opposite signs are thought to be rare, climate factors that simultaneously satisfy formulas (4) and (5) should not exist, apart from effective day length. By using effective day length, we can explain not only the north-south gradient of height and weight but also the regional divergence of height and weight in Japan.

The results of this study indicate that for equal heights, weights are greater in regions with a long day length. Moreover, for equal weights, heights are greater in the regions with a short day length. Thus, geographical differences in the heights and weights of Japanese teens can be explained by geographical differences in effective day length. These facts indicate that obesity will often occur in regions where the effective day length is long in proportion to body weight or where the effective day length is long in proportion to body height. This may explain some of the geographical differences in the prevalence of obesity. The prevalence rate of obesity for Japanese children is higher on the Pacific side of northern and southern Japan [5]. This may be because the effective day length is longer on the Pacific side of northern and southern Japan compared with the side on the Sea of Japan.

However, the impact of the effective day length on height differs depending on weight, and the impact of the effective day length on weight differs depending on height; therefore, it is impossible to anticipate the incidence of obesity only from information on the annual mean effective day length. If we know the annual mean effective day length, we can forecast body size; however, we cannot predict body composition. Based on the above, the real cause of obesity seems to lie in how day length and thyroid hormone activity are associated with body composition.

However, the elucidation of the causes of obesity is difficult. The occurrence of obesity may depend on the precise timing of light exposure during growth. Furthermore, the seasonal differences in effective day length between summer and winter may be relevant. Moreover, the effect of thyroid hormone depends on age and interplay with other hormones [14–17,24].

To the best of our knowledge, this is the only study that discusses the relationship between geographical differences in physique and day length. Recently, many studies on the relationship between day length and obesity have been published [25,26]. The concept of effective day length and the association with thyroid hormone activity may unitarily link geographical differences in physique and the relationship between day length and obesity at an individual level.

All ecological studies are likely to suffer from ecological fallacies, and this study offers only a partial explanation for the geographical differences in body size in Japanese children and adolescents. In particular, this study is based on two major assumptions: the first is that the development of the human body is affected by relatively strong natural day light, and the second is that human growth is affected by thyroid hormone activity and controlled by epigenetic mechanisms. Moreover, it is unknown whether the particular phenomena occurring in hypothalamic cells in Siberian hamsters also apply to human systemic physiological development. The physiological impact of day length on body composition is also unknown. These physiological mechanisms need to be clinically demonstrated. Therefore, additional studies using individual-level data to evaluate the impact of photoperiodic factors on anatomical factors should be pursued.

## Supporting information

S1 Fig Map of the 47 prefectures of Japan. The numbers correspond to the prefecture information presented in S1–S3 Tables.
(TIF)

S1 Table Standardized height of 5- to 17-year-old children and adolescents in each prefecture of Japan averaged over a 25-year period (1989–2013). (XLS)

S2 Table Standardized weight of 5- to 17-year-old children and adolescents in each prefecture of Japan averaged over a 25-year period (1989–2013). (XLS)

S3 Table Basic association between standardized heights and weights for each age group. (XLS)

S4 Table Population-weighted effective day length derived from Mesh Climatic Data 2000 for each prefecture of Japan. (XLS)
