# Supplementary figures and images for: Day length may make geographical difference in body size and proportions: An ecological analysis of Japanese children and adolescents
Source: PLoS One. 2019 Jan 22;14(1):e0210265. doi: 10.1371/journal.pone.0210265 (PMC6342304; doi:10.1371/journal.pone.0210265)

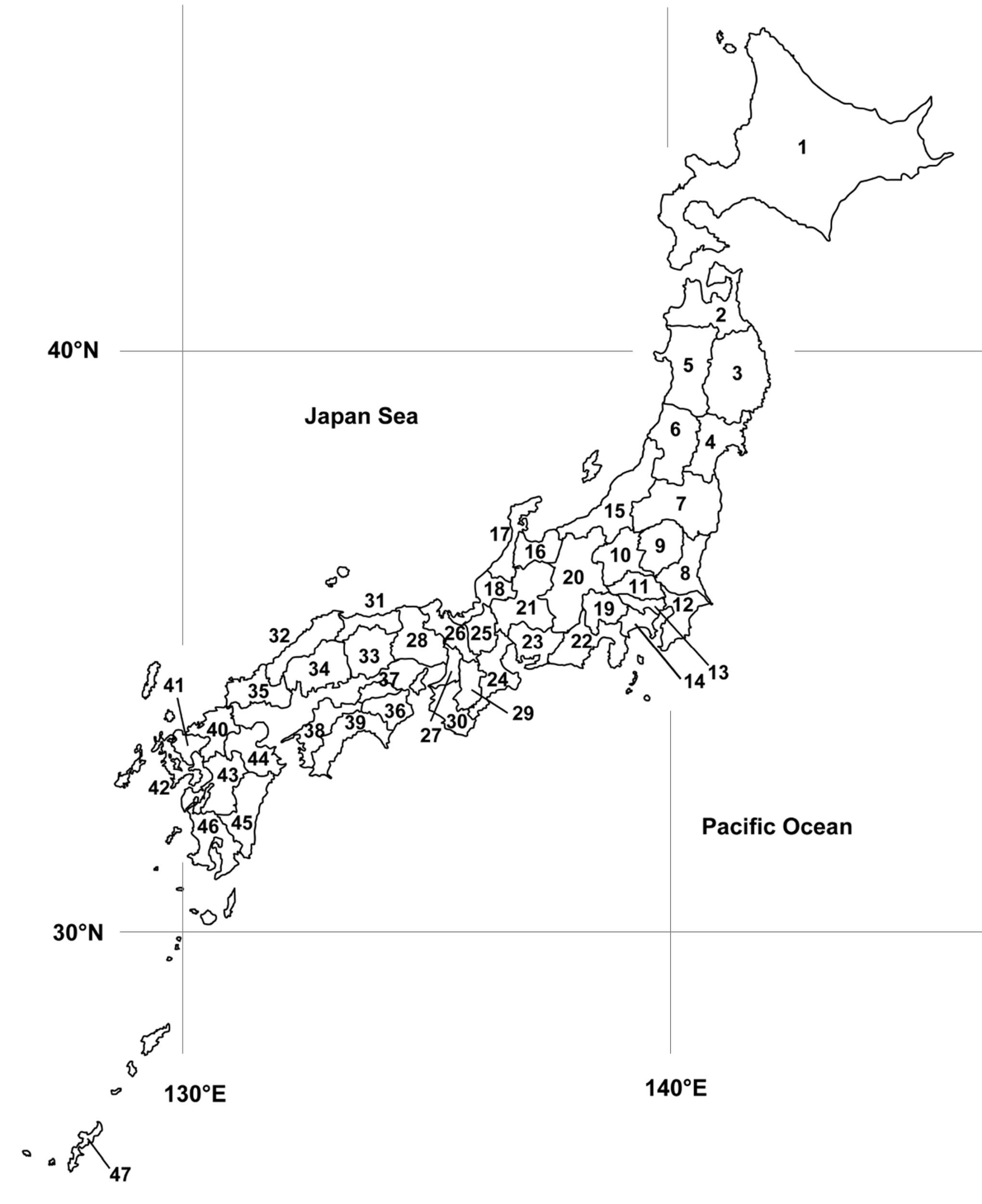

Supplement: S1 Fig — The numbers correspond to the prefecture information presented in S1–S3 Tables. (TIF) [file pone.0210265.s001.tif]
